# From innovation to integration: a global mixed-methods study of VR, metaverse, and 3D simulation in healthcare training and clinical setting

**DOI:** 10.3389/fdgth.2025.1632528

**Published:** 2025-09-08

**Authors:** Daisuke Tomita, Mohamed Abdelhakim, Julia Bartkova, Akkoyun Gulsum, Atsushi Sato, Naif H. Alshiblan Alotaibi, Mamdouh Aboulhassan, Li Dongcai, Yumiko Tomita

**Affiliations:** ^1^ Mirise Clinic, Tokyo, Japan; ^2^ Mirise Orthodontics, Tokyo, Japan; ^3^ Japan Oral Health Association, Tokyo, Japan; ^4^ Department of Plastic, Reconstructive and Aesthetic Surgery, Nippon Medical School, Tokyo, Japan; ^5^ Department of Burns and Plastic Surgery, Faculty of Medicine, Institution Shared with University Hospital Brno, Masaryk University, Brno, Czechia; ^6^ Department of Otolaryngology—Head and Neck Surgery, Al Faisal University, Riyadh, Saudi Arabia; ^7^ Department of Pediatric Surgery, Cairo University Hospital, Cairo, Egypt; ^8^ Department of Otolaryngology, Shenzhen Longgang Hospital and Shenzhen Otolaryngology, Research Institute, Shenzhen, China

**Keywords:** immersive technologies in healthcare, virtual reality, metaverse, 3D display technology, medical education, surgical training, technology adoption barriers, global digital healthcare transformation

## Abstract

**Background:**

Immersive technologies in healthcare including virtual reality (VR), metaverse platforms, and 3D display technology are transforming global healthcare by improving medical education, advancing surgical training, enhancing patient preparedness, and facilitating remote collaboration. Adoption varies regionally due to infrastructure, cost, and digital literacy gaps. This study examined their impact on healthcare training and delivery outcomes and identified key integration barriers.

**Methods:**

This mixed-methods instructional-integration study spanning four regions, Japan, the Middle East and North Africa, China, and the United States, utilized pre- and post-training surveys. Participant confidence in using immersive technologies was rated on a 5-point Likert scale. Paired t-tests determined significance. Thematic analysis of qualitative data (open-ended responses) identified key benefits and implementation challenges.

**Results:**

Of 350 healthcare professionals, 300 completed both surveys. Confidence improved significantly across all technologies: VR simulators (2.8–4.2), metaverse platforms (3.1–4.0), and 3D display systems (3.2–4.3), all *p <* 0.05. Regional trends were consistently positive, with favorable outcomes in surgical precision and spatial understanding (Cairo University, Al Faisal University). Thematic analysis cited expense (62%), limited infrastructure (56%), and need for context-specific training (49%) as key barriers; 88% of participants reported increased willingness towards applying immersive technology in healthcare settings.

**Discussion:**

Immersive technologies significantly enhance medical education and procedural training, demonstrating cross-regional applicability. Favorable feedback-based gains in user confidence underscore their transformative potential. Equitable adoption requires tackling systemic barriers through strategic investment, localized customization, and international collaboration. These findings offer actionable insights to inform policy and program development for digital healthcare transformation.

## Introduction

The digital transformation of healthcare has profoundly modified the landscape of medical education and clinical practice, particularly over the past two decades. Initially driven by advances in information technology, this shift rapidly accelerated during the COVID-19 pandemic (1). The global crisis highlighted the urgent need for remote healthcare delivery, virtual education, and efficient resource management. In response, therefore, technologies such as virtual reality (VR), augmented reality (AR), and telemedicine have since become integral to contemporary healthcare education and delivery systems (2).

Countries like Japan have spearheaded this integration by leveraging their strong foundation in technological innovation to enhance clinical workflows, surgical planning, and patient engagement strategies (3). The adoption of these digital tools, however, remains disparate across regions. While high-income countries have rapidly incorporated immersive technologies, substantive barriers, including inadequate infrastructure, limited funding, and insufficient training opportunities, continue to hinder the same in many resource-limited settings (4).

In certain parts of Africa, for example, rural areas often lack access to robust telemedicine platforms, and several countries in the Middle East continue to face delayed implementation of advanced tools such as 3D surgical planning systems (5–7). These disparities underscore the need for inclusive, targeted, and globally informed interventions and strategies to facilitate the equitable adoption of digital healthcare solutions worldwide.

### Integrating VR, metaverse, and 3D display systems into medical education and practice

Immersive technologies such as VR, metaverse platforms, and 3D display systems are among the most promising novel technologies gaining traction in modern healthcare. From advancing medical education to improving clinical outcomes and supporting patient-centered care, these tools offer promising approach in improving the delivery and engagement across a wide range of diverse clinical and educational settings.

#### Extended reality (XR) in healthcare

Extended reality (XR), encompassing virtual, augmented, and mixed reality, has rapidly expanded across clinical and educational settings. XR applications in medicine can be broadly categorized into three domains: (1) Simulation-based training platforms for healthcare professionals; (2) Immersive educational tools for patients; and (3) Therapeutic interventions. For instance, VR surgical simulator systems enable surgeons to rehearse complex procedures in risk-free environments, enhancing both technical proficiency and procedural confidence before performing actual surgical procedures (8). Similarly, VR-based physical therapy platforms have shown promise in accelerating patient rehabilitation and improving outcomes (9–11).

Table 1 outlines the key terminology used throughout the manuscript to ensure consistent and clear reference to various immersive technologies.

**Table 1:** Definitions of key terms used in the study.

Term	Definition
Immersive technology	Technologies that simulate realistic environments (e.g., VR, AR, MR)
VR (virtual reality)	Fully immersive digital simulation, typically via headset
AR (augmented reality)	Overlay of digital content onto real-world view
XR (extended reality)	Umbrella term covering VR, AR, and MR
Metaverse	Persistent, shared digital environments for interaction and simulation

#### Metaverse for telemedicine and collaboration

The metaverse extends telemedicine capabilities by introducing immersive, interactive environments for both patient consultations and interdisciplinary collaboration. Using avatars and shared virtual spaces, healthcare professionals can engage in dynamic diagnostic discussions and collaborative treatment planning. For example, a metaverse platform might facilitate real-time collaboration among a multidisciplinary team as they review a 3D model of a patient's anatomy (12).

#### 3D display systems for surgical planning and education

Three-dimensional (3D) display systems offer high-fidelity visualization of anatomical structures, robustly improving both medical education and surgical planning. Compared to traditional two-dimensional (2D) imaging, 3D systems enable medical students and clinicians to better understand complex spatial relationships and procedural nuances (13, 14). Their integration into clinical workflows is increasingly recognized as a tool for deepening anatomical comprehension, supporting preoperative decision-making, and enhancing surgical planning.

#### Implementation example: a fully digital healthcare workflow

**Figure 1:**
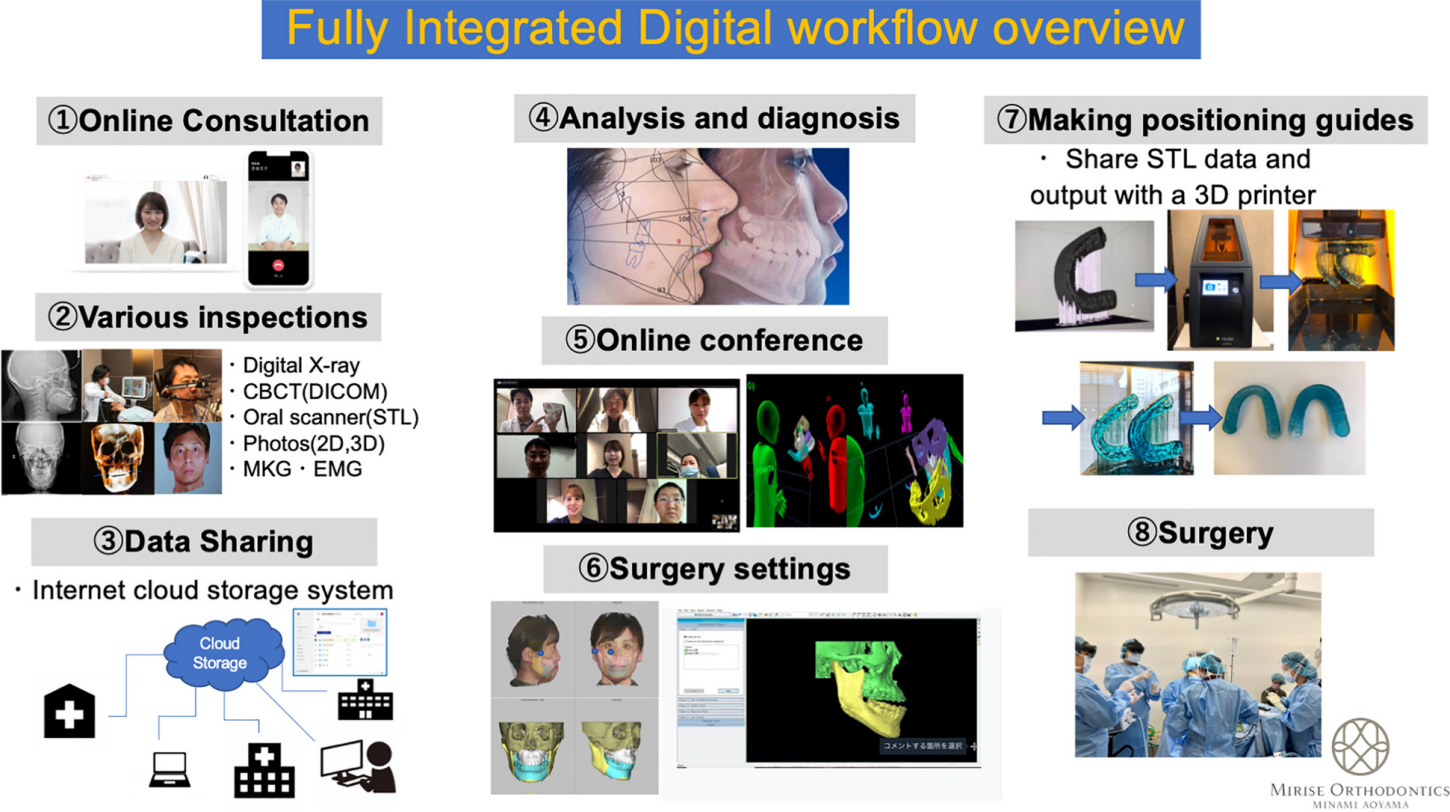
Comprehensive model of a fully digital healthcare workflow illustrating the integration of immersive and advanced technologies to streamline clinical processes, enhance patient care and engagement, and improve healthcare outcomes.

Dr. Daisuke Tomita, an experienced clinical faculty in digital surgery and digital health transformation, developed a comprehensive model for a fully digital healthcare workflow grounded in immersive and cloud-based technologies. His approach, as illustrated in Figure 1, integrates the following key components:

1. **Virtual Consultations:** Optimizing provider-patient communication through secure, cloud-based platforms.
2. **VR-based Training:** Upskilling healthcare teams *via* high-fidelity, simulation-based learning experiences.
3. **Metaverse-Based Surgical Planning:** Facilitating collaborative preoperative planning in immersive, shared virtual environments.
4. **Intraoperative Navigation:** Leveraging augmented reality (AR) to improve surgical precision in real time.
5. **Postoperative Management:** Supporting seamless follow-up care and secure data sharing for case presentations.

This model has demonstrated tangible improvements in workflow efficiency, surgical preparedness, and collaborative decision-making. It also proffers a replicable framework for broader implementation globally and across diverse healthcare settings (15). Figure 1 illustrates the digital workflow and its integration of immersive technologies across each phase of care delivery, from consultation to follow-up.

## Methods

### Study design

This study employed a mixed-methods educational implementation design to evaluate the integration and impact of VR, metaverse platforms, and 3D display systems in both medical education and clinical practice. Workshops were conducted across four global regions; Japan, the Middle East and North Africa (MENA), China, and the USA, to assess healthcare professionals' confidence in using digital tools for educational and clinical purposes.

### Participants

A total of 350 healthcare professionals participated in the workshops, including medical students, residents, practicing clinicians, and medical educators. Participants were recruited from multiple regions, including North America, Europe, the Middle East, and Asia.

Participants were recruited through institutional mailing lists, targeted email outreach, and professional social media platforms. Recruitment was open and voluntary, which may have led to a bias toward participants with a higher interest in digital tools.

All individuals voluntarily enrolled in the workshops, each of which lasted approximately 4 h. Each participant attended one workshop focused on evaluating the impact of using immersive digital healthcare technologies, including VR simulators, metaverse platforms, and 3D display systems. Pre- and post-workshop surveys were administered to assess changes in participants’ confidence levels in using these tools for educational and clinical purposes.

**Figure 2:**
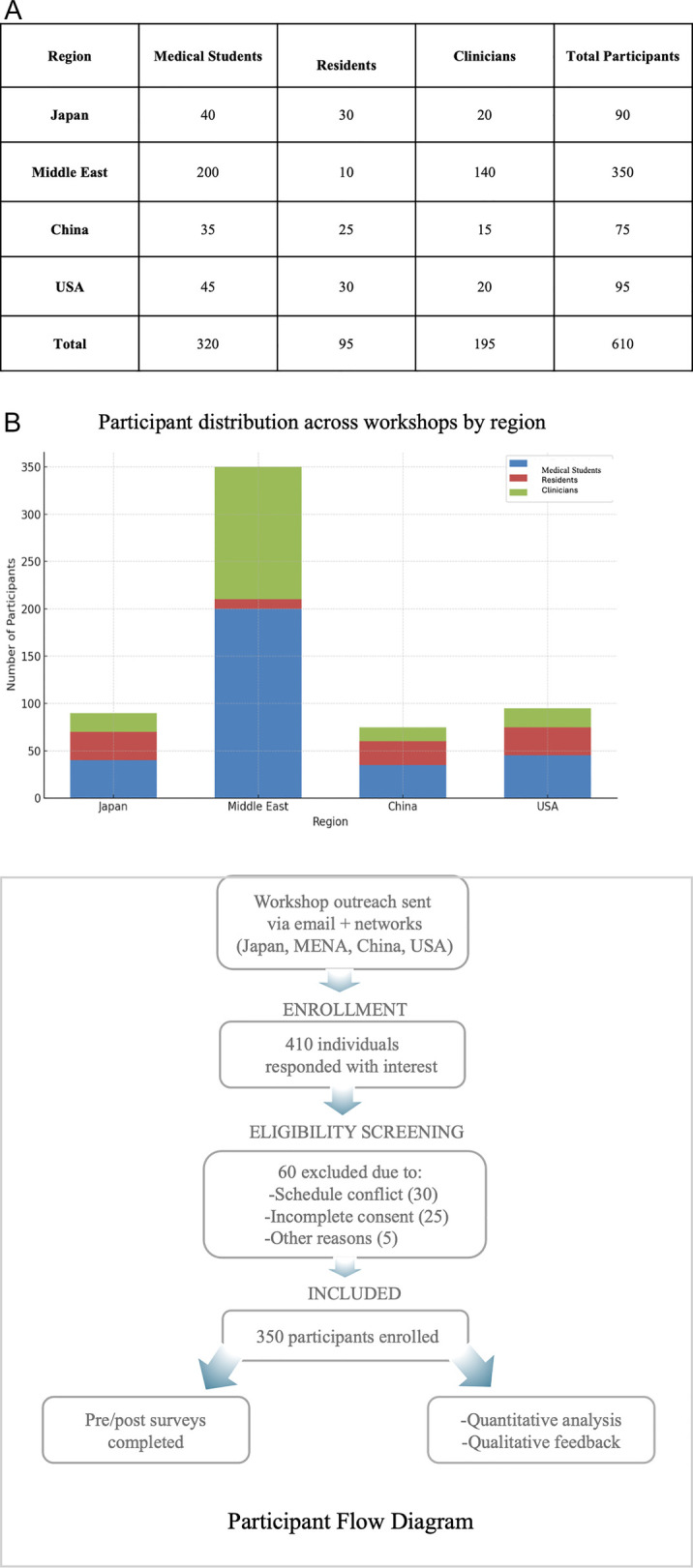
**(A)** Participant demographics across workshops (grouped bar chart) showing the distribution of medical students, residents, and clinicians by region, with a notably high proportion of student participants from the Middle East. Some participants held multiple roles and/or attended more than one regional workshop. Therefore, the cumulative count exceeds the total number of unique participants (*n* = 350). **(B)** Participant recruitment and data inclusion flow. Flowchart summarizing outreach, screening, enrollment, and final inclusion of participants across four regions. Of 410 interested individuals, 350 were enrolled; 340 completed surveys and 221 provided qualitative feedback.

A grouped bar chart (Figure 2A) summarizes participant demographics by region and professional role, highlighting a notably high proportion of student participants from the Middle East.

Some participants held multiple roles and/or attended more than one regional workshop. Therefore, the cumulative count exceeds the total number of unique participants (*n* = 350). Figure 2B showing a flowchart of the participant recruitment and data inclusion flow summarizing outreach, screening, enrollment, and final inclusion of participants across four regions.

### Intervention

**Figure 3:**
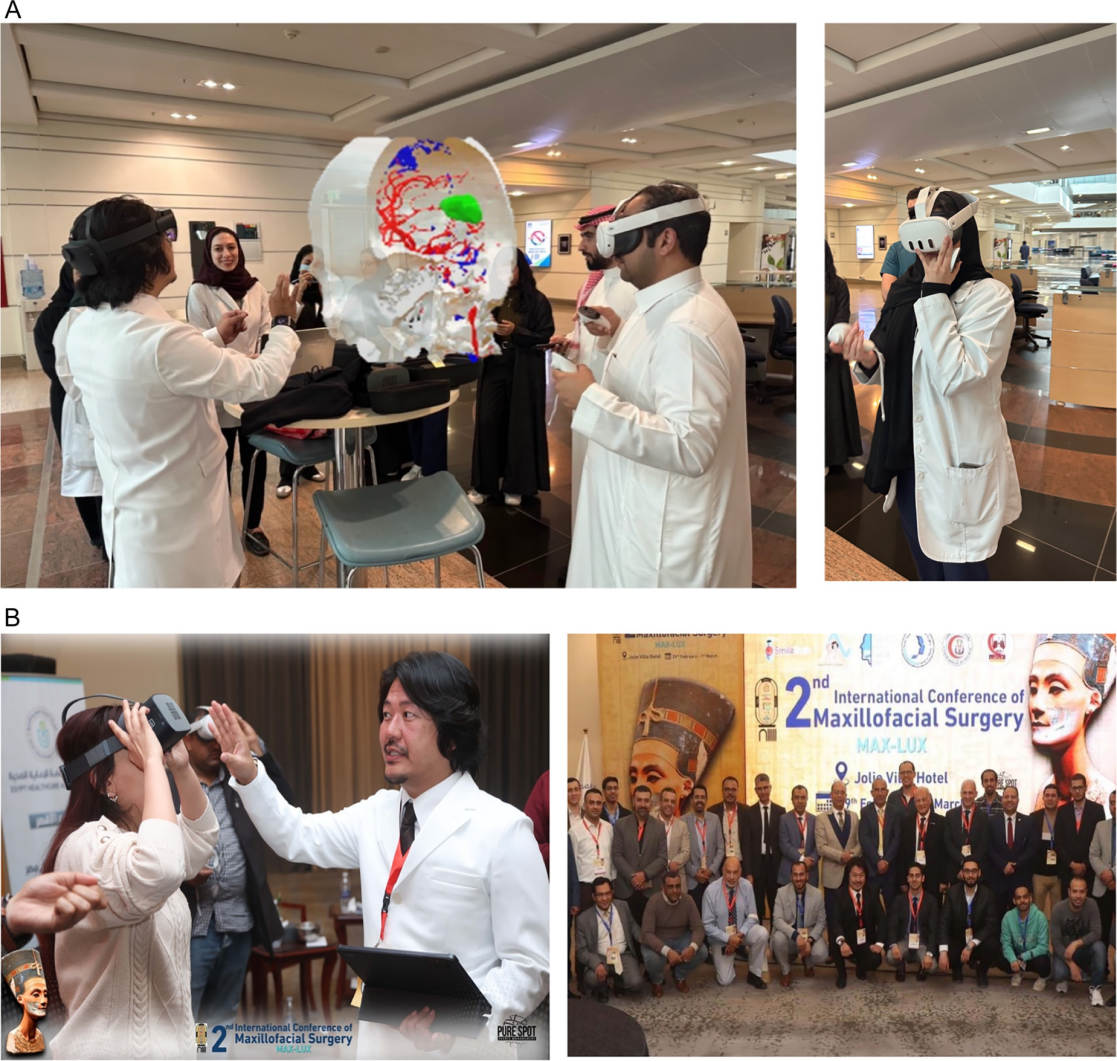
Immersive virtual reality workshops (VR) illustrating educational and clinical applications. **(A)** Medical undergraduate anatomy training at Al Faisal University, Riyadh, Saudi Arabia, with an immersive and interactive learning experience. **(B)** Surgical VR workshop for maxillofacial surgeons at a global conference sponsored by Cairo University and the Ministry of Health and Welfare in Luxor, Egypt, demonstrating advanced methods for surgical training.

The workshops provided participants with hands-on experience using these three immersive technologies: VR simulators, metaverse platforms for telemedicine, and 3D display systems for anatomical visualization (Figure 3). The VR simulators focused on surgical training, enabling participants to practice procedures in a risk-free environment. Figures 3A,B depict immersive virtual reality workshops for undergraduate anatomy training and advanced surgical education, respectively. Metaverse platforms supported virtual consultations and collaborative treatment planning, while 3D display systems facilitated deeper understanding of complex anatomical structures by enhancing spatial perception through immersive interactive visualizations.

### Data collection

Two types of data were collected: quantitative data from pre- and post-training surveys, and qualitative data from structured feedback forms.

### Quantitative data

Participants completed a structured survey before and after the workshop to assess their self-reported confidence in using each of the three technologies: VR simulators, metaverse platforms, and 3D display systems. Confidence levels were measured on a 5-point Likert scale (1 = Not confident at all, 5 = Very confident). These data were used to evaluate changes in participants' confidence levels resulting from the training intervention.

### Qualitative data

Participants also provided feedback through open-ended questions exploring their experiences with the technologies, perceived benefits, implementation challenges, and suggestions for improving training programs. Responses were analyzed thematically to identify common themes and areas for improvement regarding the adoption of digital tools.

### Ethics

The study was approved by the relevant institutional review boards of all participating countries/institutions. Participation was voluntary, and informed consent was obtained from all participants prior to enrollment. All data were anonymized at the point of collection. Any identifiable personal or clinical data was recorded only with clear informed consent.

## Statistical analysis

### Quantitative data analysis

Descriptive statistics were used to summarize participants' demographic characteristics and pre- and post-training confidence scores. Confidence scores were computed for each of the three technologies—VR simulators, metaverse platforms, and 3D display systems—and stratified by region. Paired sample *t*-tests were performed to compare pre- and post-training confidence scores for each technology and region, assessing the statistical significance of changes in confidence levels. A significance threshold of *p* < 0.05 was used to determine whether the observed changes were statistically significant.

Additionally, for each region, confidence scores were compared across the three technologies to examine differences in the perceived impact of each technology on participants' confidence. All statistical analyses were performed using SPSS v26 (IBM Corp., Armonk, NY, USA).

### Qualitative data analysis

Qualitative feedback was analyzed using thematic analysis guided by a grounded theory approach. Open-ended responses were independently reviewed and coded by two researchers to identify recurring themes related to the perceived benefits, challenges, and barriers associated with adopting digital healthcare technologies. Initial codes, such as “increased patient understanding via visualization,” were generated inductively. Discrepancies between coders were resolved through consensus to ensure consistency and maintain inter-rater reliability. The interrater reliability, measured using Cohen's kappa, was 0.79, indicating substantial agreement. Emergent themes were categorized and further analyzed in relation to participants' reported confidence changes and perceived effectiveness of the technologies.

### Reliability and validity

The reliability of the findings was ensured by cross-checking the data and resolving any inconsistencies between the quantitative and qualitative analyses. The validity of the pre- and post-training surveys was established through pilot testing with a small group of healthcare professionals prior to the main study. Feedback from the pilot group informed adjustments to the survey questions to improve clarity, relevance, and alignment with the study objectives.

## Results

### Regional workshop outcomes

#### Participant feedback and impact on research motivation

**Figure 4:**
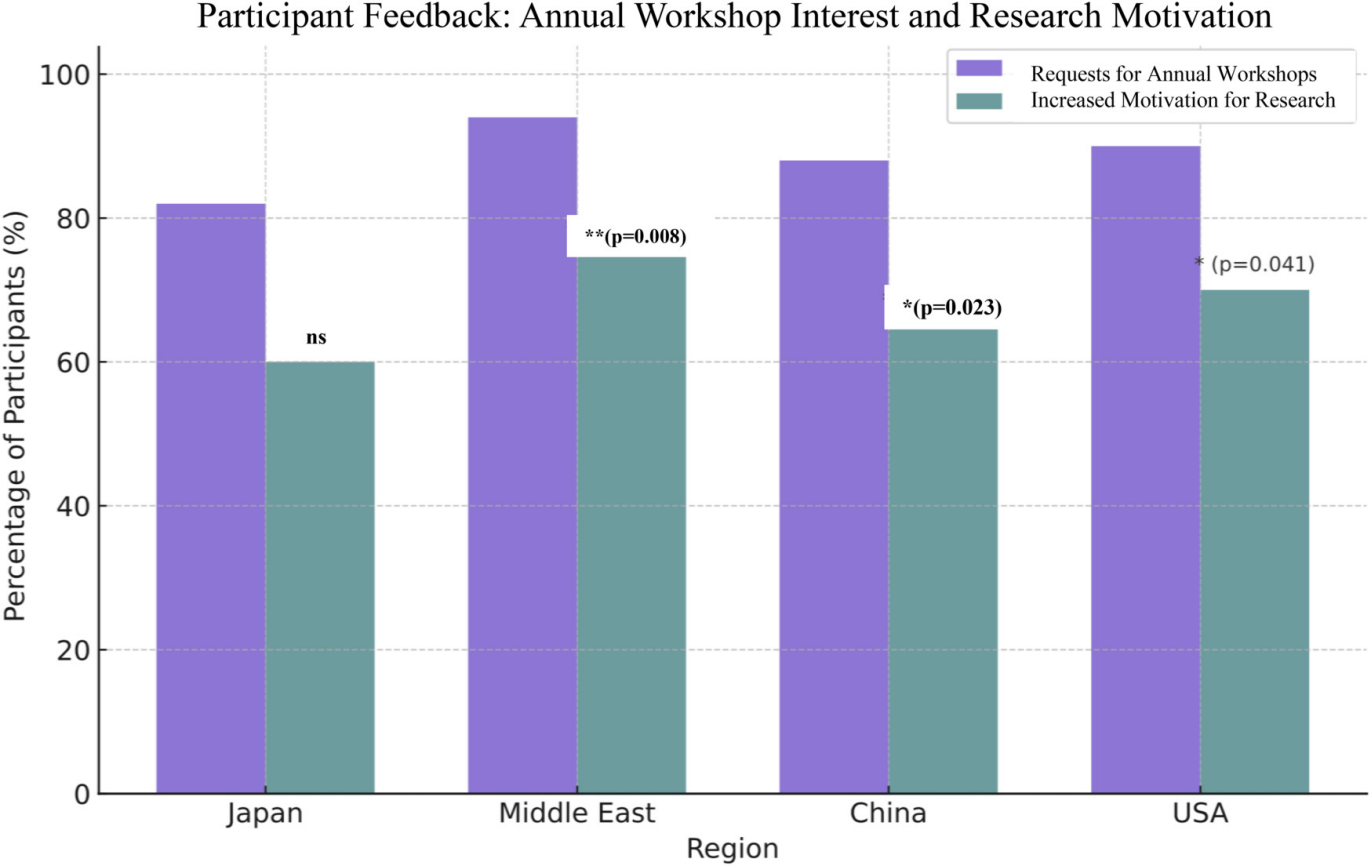
Post-workshop survey responses indicating interest in annual immersive training workshops and the motivation to pursue further research. The data reflect strong positive feedback across all regions, with particularly high levels of enthusiasm reported in the Middle East and the United States.

Participant feedback from the workshops revealed high demand for continued annual training and strong motivation to further engage in digital health research across all surveyed regions. Notably, over 90% of participants in the Middle East and the tech expressed interest in recurring annual workshops, significantly higher than participants from Japan (82%) and China (88%). The motivation to pursue related research showed a similar trend with the highest proportions in the Middle East (75%) and the USA (70%), followed by China (65%) and Japan (60%). These findings presented in (Figure 4), highlight regional enthusiasm for integrating digital tools into clinical education and research, as shown by strong positive feedback across all regions. Post-workshop survey responses indicated strong participant interest in recurring immersive training and increased motivation to pursue related research. Chi-square tests confirmed the statistical significance (*p* < 0.05) of regional differences in workshop demand and research motivation.

The regional workshops highlighted diverse advancements and challenges in digital healthcare adoption across the Middle East, the United States, and China. In the Middle East, collaborations with institutions such as Al Faisal University and Cairo University showcased strong alignment with national visions like Saudi Arabia's Vision 2030. Workshops focused on VR, 3D displays, and metaverse applications were well received, particularly in surgical departments, though infrastructure limitations and evolving regulatory frameworks continue to pose challenges for widespread implementation. In the U.S., Stanford University played a pivotal role in leading global digital transformation through its IT Technology Training, offering hands-on, AI-integrated curricula under expert guidance, and fostering interdisciplinary collaboration with international partners. Meanwhile, in China, Shenzhen Longgang District People's Hospital served as a model for integrating telemedicine, AI diagnostics, and VR training, with workshops significantly boosting exposure and collaboration between Chinese and global stakeholders (16). Collectively, these regional efforts underscore the growing global momentum toward digitally enhanced medical education and clinical practice.

### Confidence gains following workshop participation

**Figure 5:**
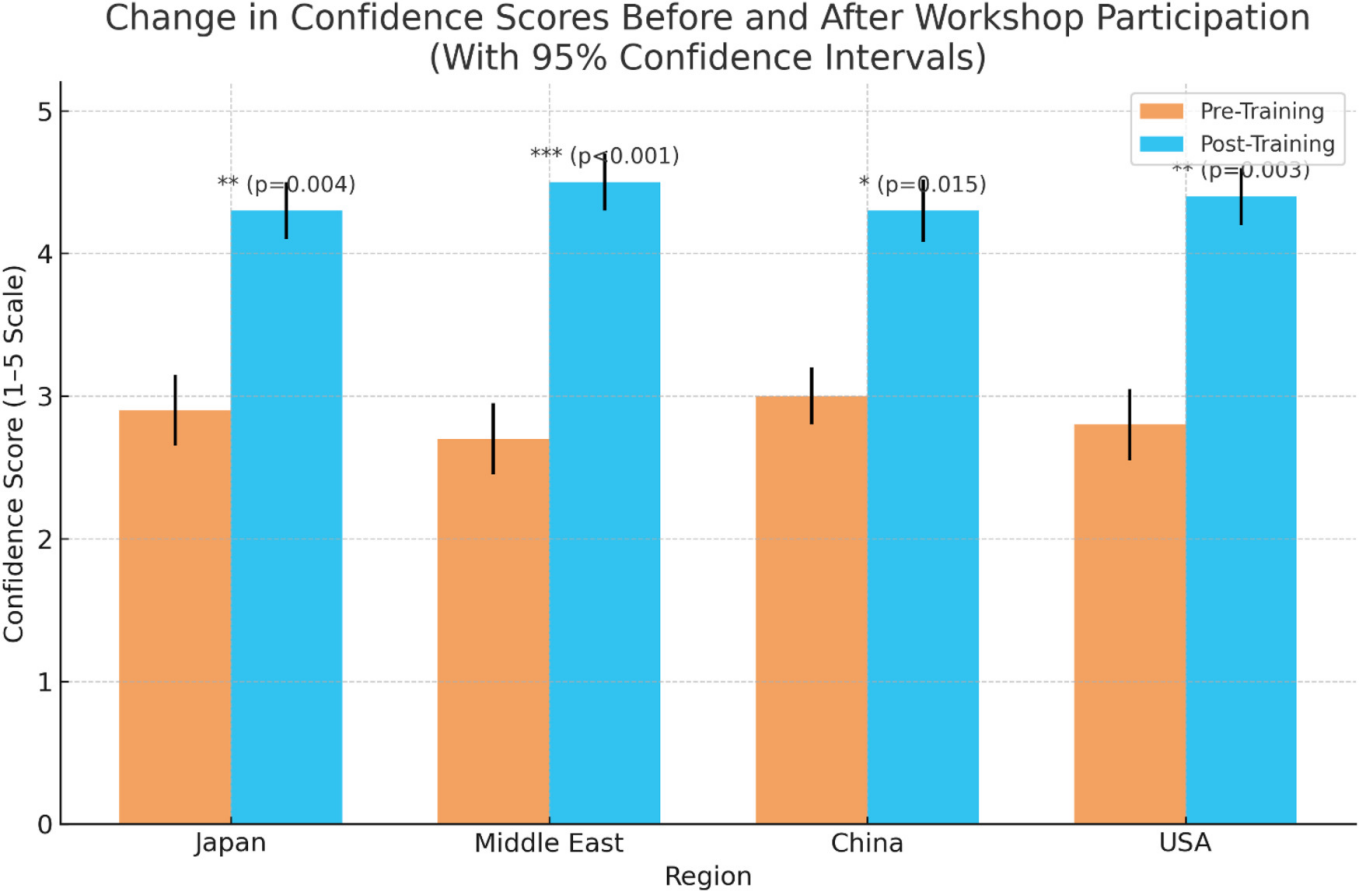
Bar chart depicting comparative pre- and post-training confidence scores across regions. The chart clearly illustrates self-assessed improvement in participant confidence following the immersive technology workshops.

Workshops significantly improved participants' confidence in using digital healthcare tools, as measured on a 5-point Likert scale. Demonstrable improvements from pre- to post-training were noted in all regions. The greatest gains, shown by rising confidence scores, were observed among participants from the Middle East (2.7–4.5) and the USA (2.8–4.4), who reported the highest improvements, followed by Japan (2.9–4.3) and China (3.0–4.2). All changes were statistically significant (*p* < 0.001). Figure 5 presents a bar chart of pre- and post-training confidence scores across regions, indicating measurable improvement following the workshops. These findings demonstrate the effectiveness of immersive, hands-on training formats in building user confidence across diverse healthcare education and service delivery environments.

### Thematic findings across regions

**Figure 6:**
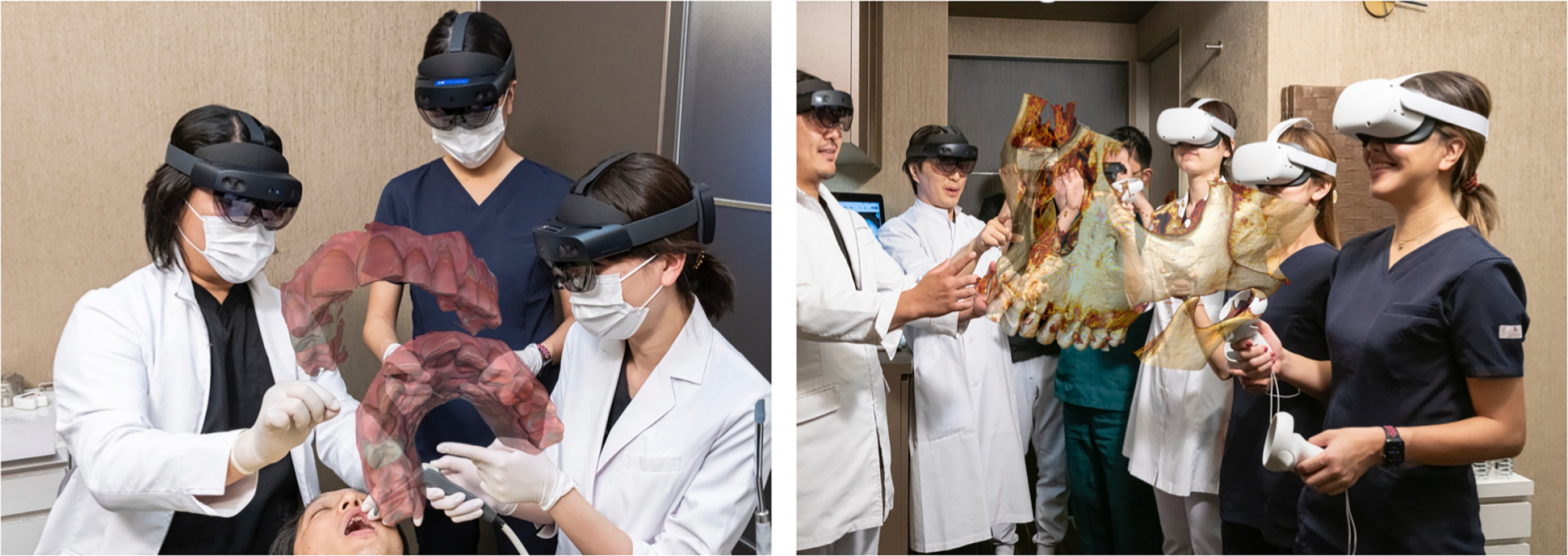
Use of virtual reality and simulation models for clinical staff training and professional development. This hands-on approach highlights innovative methods in medical education and on-the-job learning.

**Figure 7:**
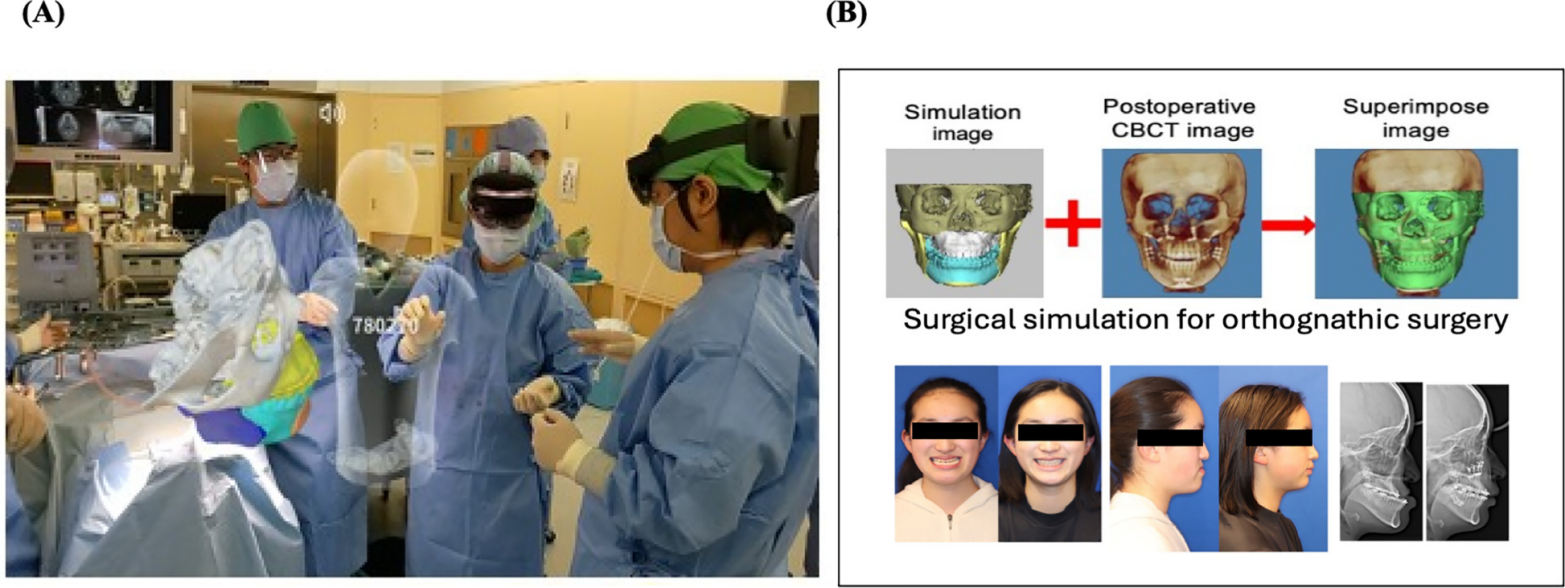
**(A)** Integration of extended reality technology into maxillofacial surgery by the authors (surgical team) at their clinic, demonstrating the clinical application of immersive technology tools in a real-world clinical setting. **(B)** Surgical simulation for orthognathic surgery using digital healthcare tools demonstrated enhanced precision, skill acquisition, and training efficiency with notably improved and favorable outcomes.

**Figure 8:**
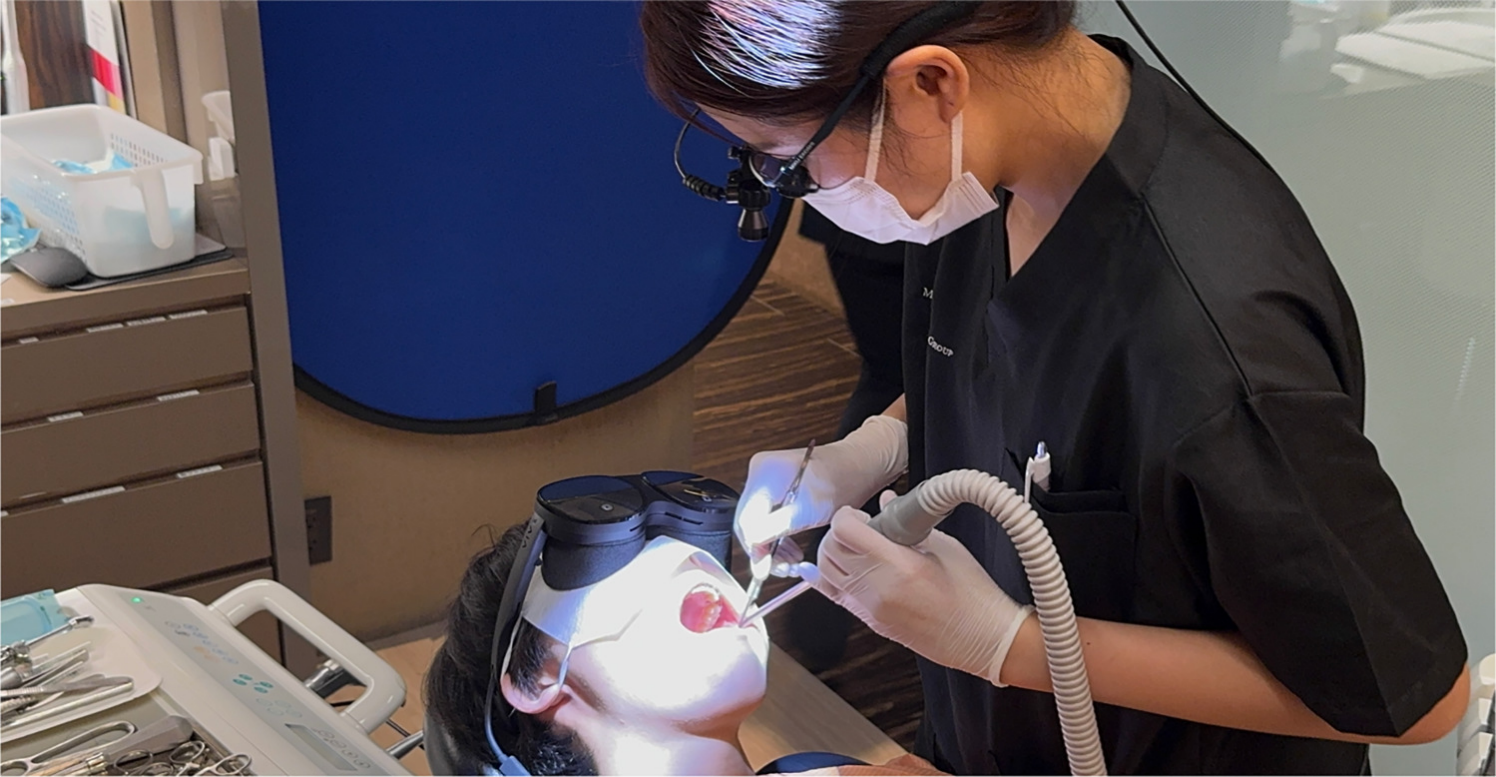
Use of virtual reality to reduce pain and alleviate anxiety during lengthy dental procedures. This application highlights the potential of immersive technology to enhance patient comfort and clinical experience.

Thematic analysis of open-ended feedback and responses revealed several recurring themes across all four regions. Participants consistently reported that VR-based training markedly enhanced surgical planning, procedural confidence, and technical performance. Figure 6 illustrates one such hands-on simulation approach used to support clinical staff training and on-the-job learning/professional development. As further exemplified in Figure 7, the integration of extended reality into maxillofacial surgery by the authors' surgical team considerably improved precision and training outcomes. Realistic simulations enabled healthcare providers to rehearse complex procedures, ultimately leading to perceived measurable improvements in both accuracy and efficiency. For patient-facing applications, immersive VR environments notably alleviated anxiety, particularly in pediatric and burn care settings, by providing calming, gamified, or interactive experiences that prepared patients emotionally for clinical interventions (17–19). The use of VR to reduce pain and anxiety during lengthy dental procedures (Figure 8) improved patient comfort and overall clinical experience. Additionally, VR-supported informed consent processes were associated with improved patient understanding and satisfaction (20–22). These thematic trends, underscored by the aforementioned statistically significant confidence gains, emphasize the broad applicability and clinical relevance of immersive digital tools in education, procedural planning, and patient engagement (Figures 6–8).

Further analysis of open-ended responses from 290 participants revealed deeper insights into the perceived benefits, challenges, and barriers to technology adoption. The most frequently cited benefits included improved spatial understanding (72%), increased procedural confidence (68%), and enhanced preparedness for complex interventions (63%). These qualitative findings aligned with the quantitative results and reinforced the reported confidence gains across regions.

Despite these benefits, participants also identified several barriers to adoption, including high implementation costs (62%), limited infrastructure (56%), lack of localized training content (49%), cultural or professional resistance (38%), and challenges in integrating digital tools into existing medical systems (34%). These concerns were consistent across regions and were perceived as key obstacles to sustainable, long-term adoption.

**Figure 9:**
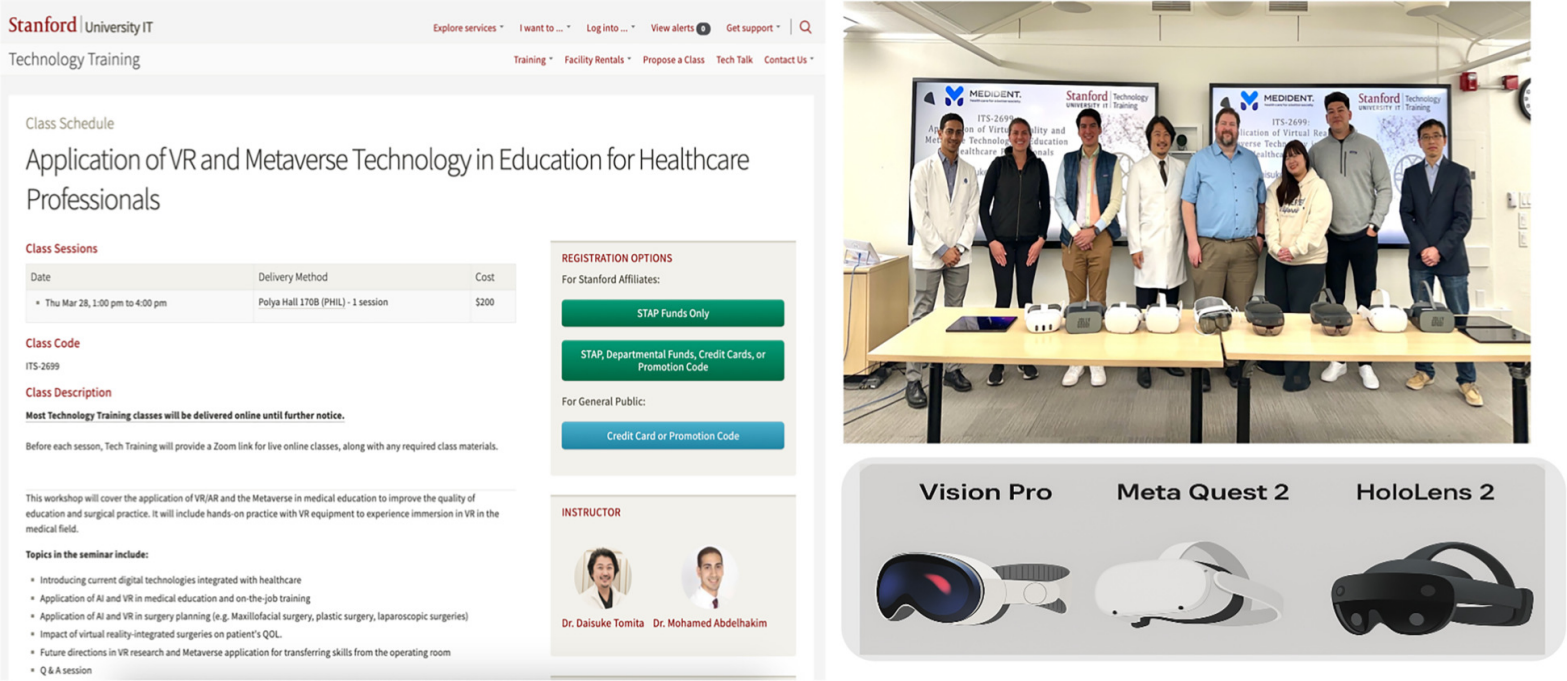
Stanford technology training classes providing hands-on experience in advanced digital technology tools to promote innovation in medical education and clinical practice. Screenshot from: https://uit.stanford.edu/service/techtraining/sessions, Stanford University.

Participants proposed several improvement strategies to address these limitations, including expanding access to affordable devices, fostering collaboration between technology developers and medical educators, and adapting training modules to regional languages and cultural contexts. Furthermore, participants emphasized the value of structured, hands-on training in building digital confidence and competence. Programs such as the Sanford Technology Training program (Figure 9) were highlighted as a model for hands-on digital skill development, impactfully fostering immersive, skill-based learning environments that support innovation in medical education (23).

These insights reiterate the importance of localized and inclusive approaches to successfully expanding and implementing immersive healthcare technologies on a global scale.

## Discussion

**Figure 10:**
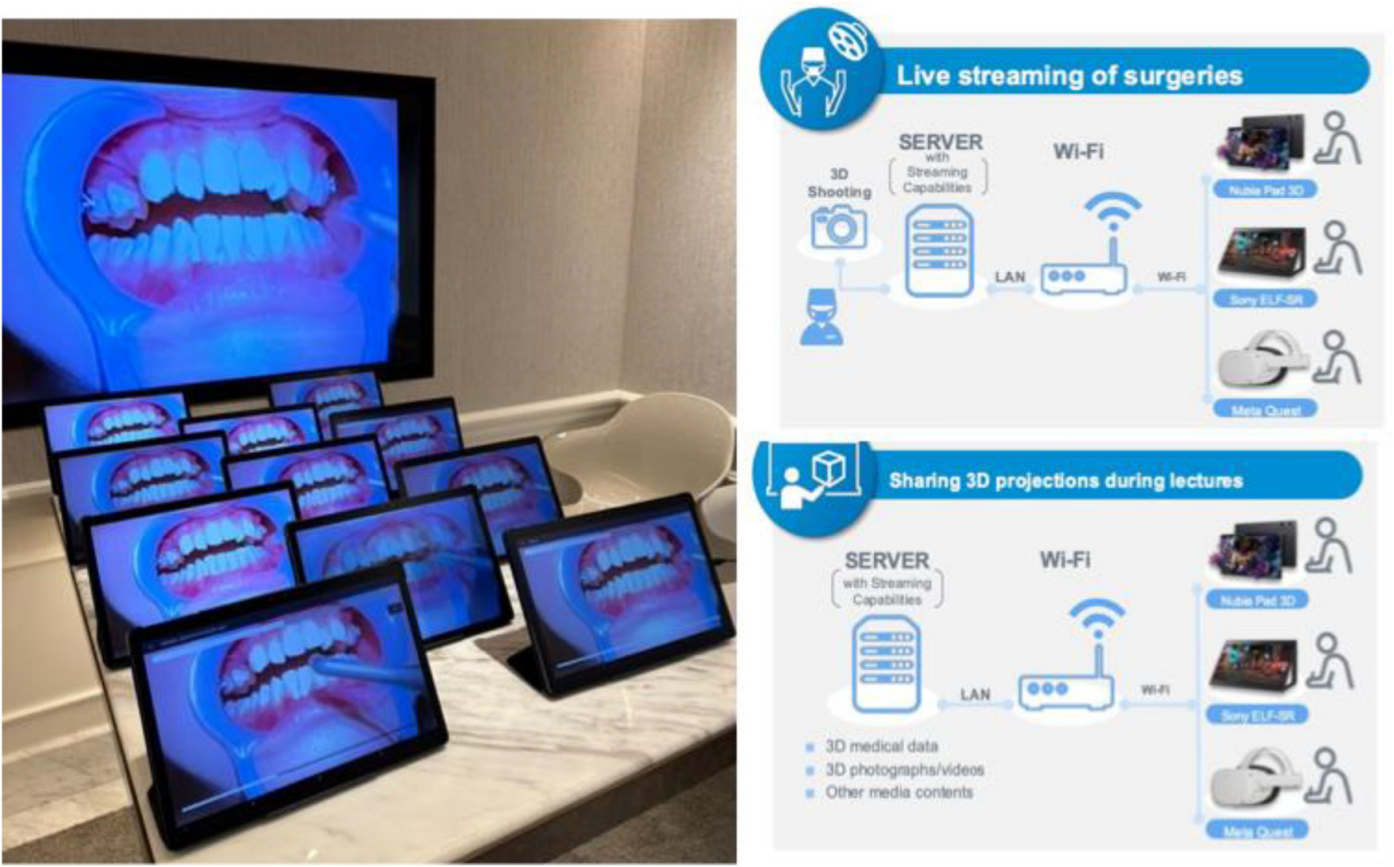
Application of a 3D display system developed in the authors’ clinic, enhancing surgical training by enabling interactive engagement with surgical procedures from multiple angles and depths, with simultaneous real-time instructor guidance.

The integration of immersive technologies into medical education and patient care demonstrates substantial potential across diverse medical education and healthcare delivery settings. The multi-regional workshops conducted in MENA, Asia, and the United States noted consistent improvements in both participants' confidence and clinical preparedness, further supporting the utility of VR and related tools in global healthcare education initiatives. These findings are consistent with existing literature that supports simulation-based learning as an effective strategy for enhancing spatial awareness, procedural accuracy, and skill retention among healthcare professionals (1–3). Figure 10 features a 3D display system developed by the authors and used at their clinic, enabling interactive surgical training with real-time instructor guidance.

In addition to these educational benefits, VR interventions effectively addressed patient-centered needs. Preoperative VR walkthroughs were associated with increased patient confidence, while pediatric applications, including interactive simulations and distraction therapy, helped reduce procedural anxiety and promote emotional resilience (24–26). These outcomes corroborate previous research suggesting that immersive technologies can serve a dual role in improving both clinician performance and patient experiences (4, 5).

The metaverse also emerged as a valuable medium for telemedicine and international collaboration (27–30).

**Figure 11:**
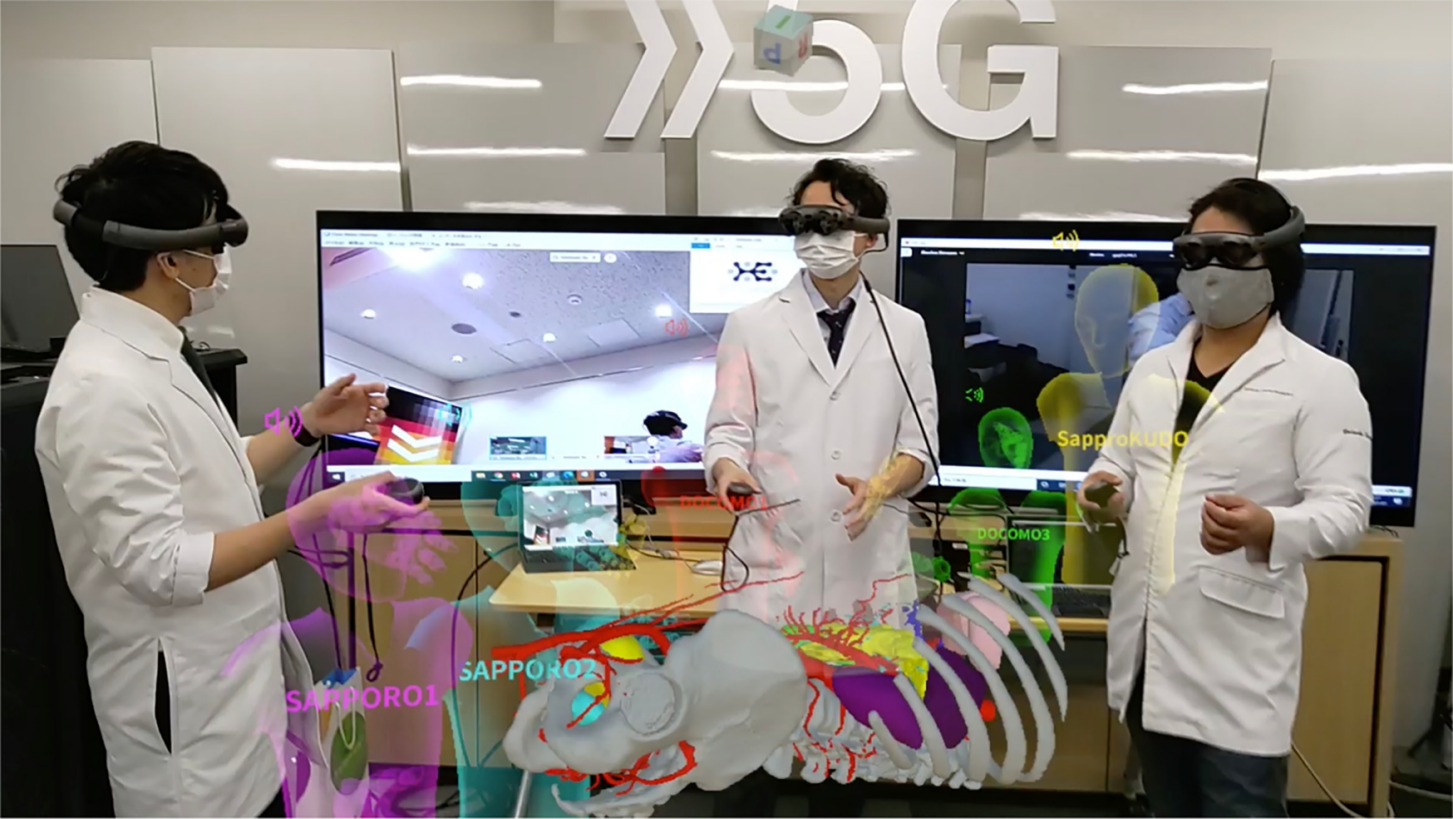
Case study on a 5G-enabled online metaverse conference connecting surgeons across different regions in Japan, demonstrating the potency of advanced connectivity in facilitating remote collaboration.

Notably, the use of 5G-enabled metaverse platforms in Japan facilitated real-time surgical discussions and 3D model interactions among geographically dispersed surgical teams. Figure 11 shows a 5G-enabled online metaverse conference that connected surgeons across multiple regions in Japan, demonstrating the role of advanced connectivity in facilitating remote surgical collaboration (6). However, disparities in infrastructure, particularly in rural or underserved areas, highlight the need for equitable access to digital health resources.

Despite these encouraging outcomes, several barriers to widespread adoption were identified. These included cultural hesitancy toward substituting face-to-face care with digital tools, the complexity of integrating immersive technologies into existing healthcare workflows, and prohibitive implementation costs (31). Nevertheless, these challenges may be mitigated through context-sensitive strategies such as localizing success stories, fostering public-private partnerships, and developing scalable training models to reduce financial burden (7–9).

**Figure 12:**
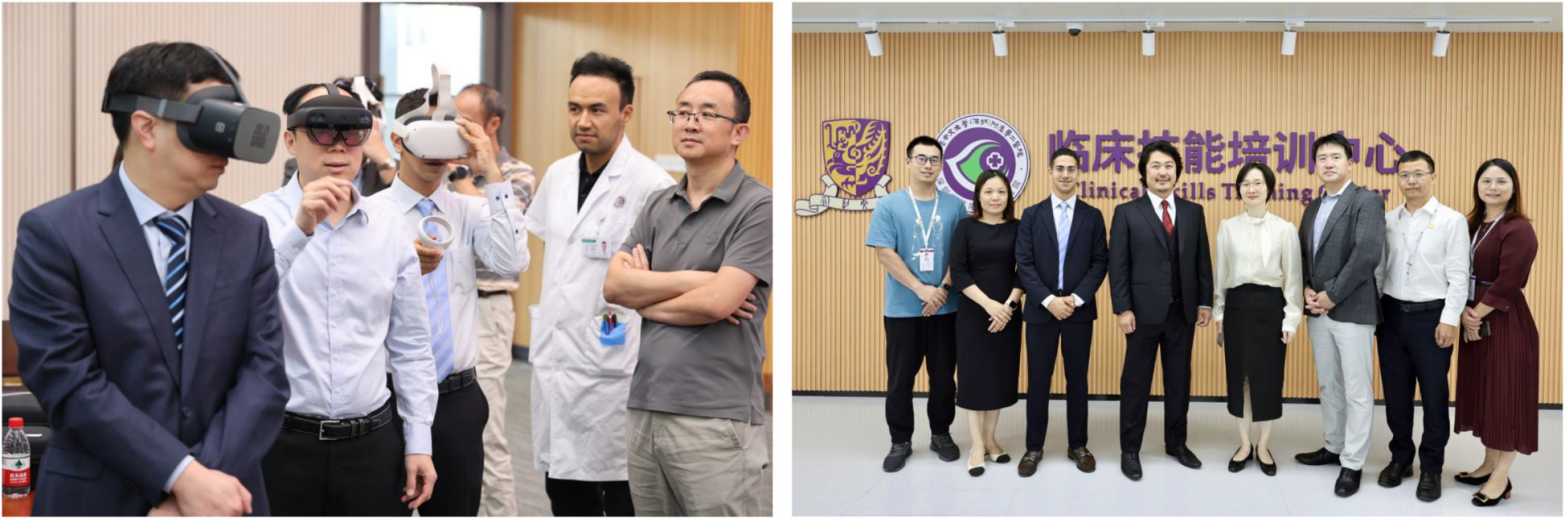
Hands-on digital transformation workshop conducted at the clinical skills training center, Longgang district hospital, 2nd affiliated hospital of the University of Hong Kong, Shenzhen, China, focusing on the integration of digital tools into clinical practice.

Workshops in diverse healthcare environments demonstrated that immersive digital training is feasible and adaptable across regions with varying levels of infrastructure. For example, the hands-on digital transformation workshop held at the Clinical Skills Training Center, Longgang District Hospital, 2nd Affiliated Hospital of the University of Hong Kong, Shenzhen, China (16), illustrates how practical integration of digital tools can be achieved into clinical practice (Figure 12).

Overall, these findings suggest that when strategically implemented, immersive technologies have the potential to transform both medical education and clinical care. Future efforts should prioritize long-term sustainability, inclusive design, and supportive policy frameworks to ensure equitable digital transformation across global healthcare systems.

### Limitations

While this study provides valuable insights into the implementation and impact of immersive technologies in healthcare education and clinical practice across multiple regions, several limitations should be acknowledged.

First, the workshops were conducted within select academic and clinical institutions, which may not comprehensively represent broader healthcare settings, particularly those in rural or resource-constrained settings. This may limit the generalizability of the findings. Second, although pre- and post-workshop surveys were used to assess changes in confidence and perceived preparedness, these self-reported measures are inherently subjective and may have been influenced by participant expectations or favorable disposition toward novel technologies. Although self-reported confidence is a widely used proxy for perceived competence, it does not directly measure objective performance or knowledge acquisition. Future studies should incorporate more robust evaluation metrics such as skill-based simulation scores or pre/post knowledge tests to validate training effectiveness.

Third, persistent disparities in infrastructure and variable access to advanced technologies across regions may have influenced both the scope and depth of training experiences. Furthermore, smaller sample sizes in some workshops limited the capacity for detailed granular subgroup analyses. Finally, the rapid evolution of immersive technologies, including VR and metaverse platforms, presents a moving target for evaluation. As hardware, software, and digital literacy continue to evolve, future studies will need to reassess effectiveness, adoption barriers, and integration strategies within real-time clinical settings.

Since the workshops were designed to introduce immersive tools and highlight successful use cases, the content may have unintentionally emphasized advantages over challenges. This could have further amplified participant enthusiasm and shaped their responses. Future programs should present a more balanced view, incorporating both benefits and adoption barriers.

Despite these limitations, the multi-country scope, diverse participant backgrounds, and consistent positive outcomes reinforce the potential for immersive technologies to play a transformative role in global healthcare education and delivery.

## Conclusion

This global mixed-methods study demonstrates that immersive technologies, encompassing virtual reality, metaverse platforms, and 3D visualization, can significantly enhance professional healthcare training, clinical practice, and patient preparedness. Participants across diverse geographic and cultural regions consistently reported increased confidence, improved surgical planning skills, and greater inclination to integrate digital tools into clinical practice.

The statistically significant gains observed across all workshop sites support the broad applicability and effectiveness of these technologies. Harnessing their full potential, however, will require strategic investment in infrastructure, development of localized implementation frameworks, and sustained institutional collaboration. Addressing persistent barriers, such as high costs, cultural resistance, and technological limitations, is essential to ensuring equitable integration across healthcare systems (32).

## Data Availability

The original contributions presented in the study are included in the article/Supplementary Material, further inquiries can be directed to the corresponding author.

## Future directions

The future of digital healthcare will be shaped by the convergence of emerging technologies; wearables for real-time monitoring, 3D-printed patient-specific models for surgical planning, and haptic-enhanced VR/AR for immersive training. AI will personalize education and support clinical decisions, while blockchain will secure data sharing across platforms. These tools, integrated into interoperable systems, will enhance precision, safety, and efficiency in both training and care. To realize this potential globally, fostering cross-border collaborations and developing culturally adaptive digital ecosystems will be key to ensuring inclusive, scalable, and sustainable innovation in healthcare.
